# iHyd-PseAAC: Predicting Hydroxyproline and Hydroxylysine in Proteins by Incorporating Dipeptide Position-Specific Propensity into Pseudo Amino Acid Composition

**DOI:** 10.3390/ijms15057594

**Published:** 2014-05-05

**Authors:** Yan Xu, Xin Wen, Xiao-Jian Shao, Nai-Yang Deng, Kuo-Chen Chou

**Affiliations:** 1 Department of Information and Computer Science, University of Science and Technology Beijing, Beijing 100083, China; E-Mail: wenxinfairy@gmail.com; 2 Department of Mathematics and Information Science, Binzhou University, Binzhou 256603, China; E-Mail: shaoxiaojian@gmail.com; 3 College of Science, China Agricultural University, Beijing 100083, China; E-Mail: dengnaiyang@cau.edu.cn; 4 Center of Excellence in Genomic Medicine Research (CEGMR), King Abdulaziz University, Jeddah 21589, Saudi Arabia; E-Mail: kcchou@gordonlifescience.org; 5 Gordon Life Science Institute, Boston, MA 02478, USA

**Keywords:** PTMs, HyP, HyL, PseAAC, discriminant function algorithm

## Abstract

Post-translational modifications (PTMs) play crucial roles in various cell functions and biological processes. Protein hydroxylation is one type of PTM that usually occurs at the sites of proline and lysine. Given an uncharacterized protein sequence, which site of its Pro (or Lys) can be hydroxylated and which site cannot? This is a challenging problem, not only for in-depth understanding of the hydroxylation mechanism, but also for drug development, because protein hydroxylation is closely relevant to major diseases, such as stomach and lung cancers. With the avalanche of protein sequences generated in the post-genomic age, it is highly desired to develop computational methods to address this problem. In view of this, a new predictor called “iHyd-PseAAC” (identify hydroxylation by pseudo amino acid composition) was proposed by incorporating the dipeptide position-specific propensity into the general form of pseudo amino acid composition. It was demonstrated by rigorous cross-validation tests on stringent benchmark datasets that the new predictor is quite promising and may become a useful high throughput tool in this area. A user-friendly web-server for iHyd-PseAAC is accessible at http://app.aporc.org/iHyd-PseAAC/. Furthermore, for the convenience of the majority of experimental scientists, a step-by-step guide on how to use the web-server is given. Users can easily obtain their desired results by following these steps without the need of understanding the complicated mathematical equations presented in this paper just for its integrity.

## 1. Introduction

**Figure 1.**
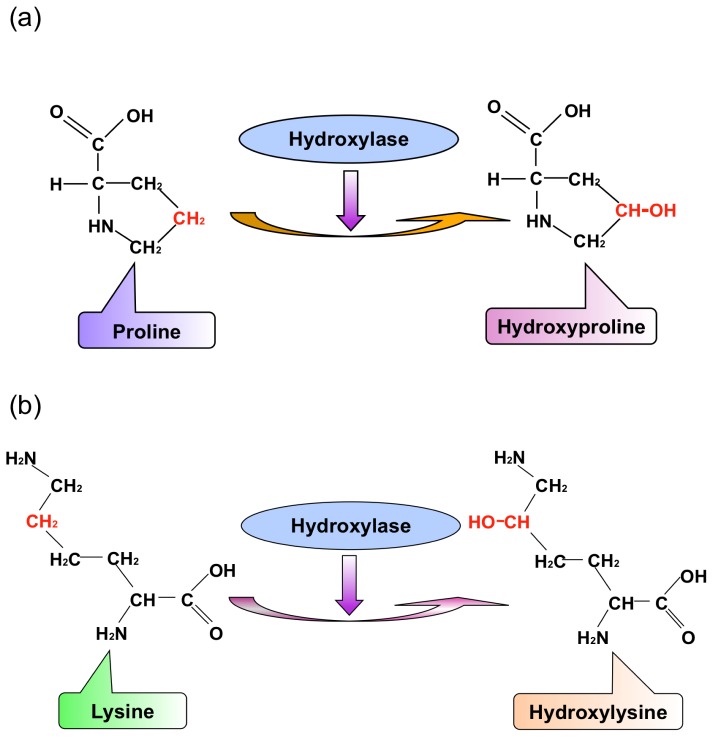
Schematic drawing to show protein hydroxylation occurring at (**a**) proline and (**b**) lysine to form hydroxyproline (HyP) and hydroxylysine (HyL), respectively.

Most proteins perform their functions after post-translational modifications (PTMs). Protein hydroxylation is one type of PTM that involves the conversion of a CH group into a COH group (Figure 1) and is closely relevant to the regulation of the transcription factor (hypoxia-inducible factor) [1]. Both the proline and lysine residues in proteins can be hydroxylated, forming hydroxyproline (Figure 1a) or HyP and hydroxylysine (Figure 1b) or HyL, respectively. However, the former is more common than the latter [2,3]. Furthermore, HyP is the key factor in stabilizing collagens [4,5], whose instability or abnormal activity may cause stomach cancer [6] and lung cancer [7,8]. Therefore, identifying the HyP and HyL sites in proteins may provide useful information for both biomedical research and drug development.

Identification of hydroxylation residues with experiments was mainly done by means of mass spectrometry [1,9], which was expensive and laborious. Facing the avalanche of protein sequences generated in the post genomic age, it is highly demanded to develop a computational method for timely and effectively identifying the hydroxylation residues in proteins. However, to our best knowledge, so far, only two papers have been published in this regard [10,11]. Additionally, further development in this important area is definitely needed for the following reasons. First, with a rapidly growing database in protein hydroxylation, the benchmark datasets used in the two methods definitely need to be updated; Second, some sequence order effects were missed, which would certainly affect their prediction quality; Third, none of them provided a publicly accessible web-server, and hence, their practical usage value is substantially limited.

The present study was devoted to develop a new predictor for identifying hydroxyproline and hydroxylysine in proteins by considering the above three aspects. The principle was based on a window sliding strategy, quite similar to the popular approach developed by Garnier and Robson [12] for predicting the secondary structure of globular proteins.

As demonstrated by a series of recent publications [13–20] and summarized in a comprehensive review [21], to develop a really useful predictor for a protein or peptide system, we need to go through the following five steps: (1) select or construct a valid benchmark dataset to train and test the predictor; (2) represent the protein or peptide samples with an effective formulation that can truly reflect their intrinsic correlation with the target to be predicted; (3) introduce or develop a powerful algorithm or operation engine to conduct the prediction; (4) properly perform cross-validation tests to objectively evaluate the anticipated prediction accuracy; (5) establish a user-friendly web-server for the predictor that is accessible to the public. Below, let us elaborate on how to deal with these five steps.

## 2. Results and Discussion

### 2.1. Benchmark Dataset

**Figure 2.**
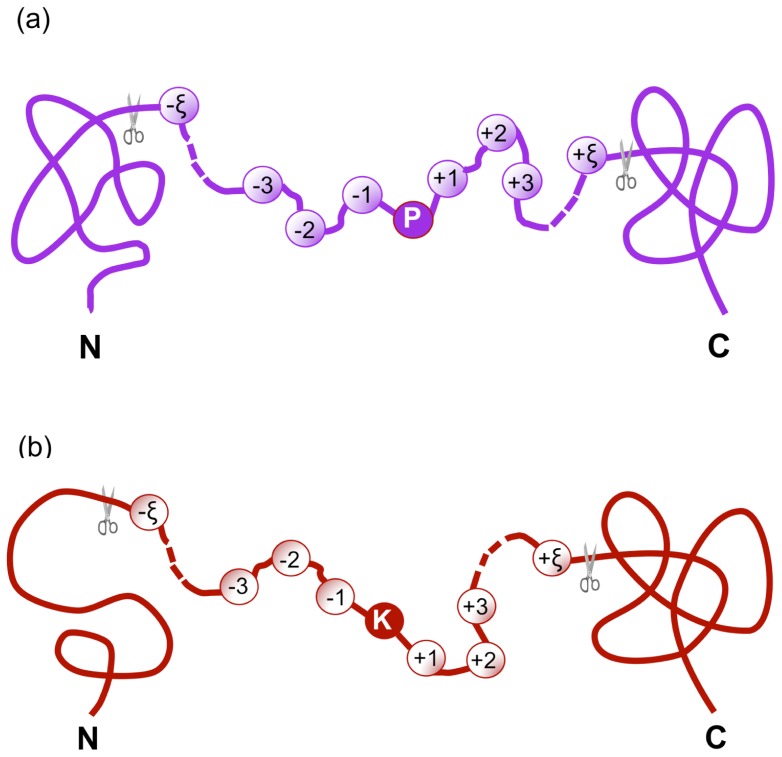
An illustration to show Chou’s scheme for peptides with (2*ξ* + 1) residues and their centers being **(a)** proline and (**b**) lysine. Adapted from Chou [27,29] with permission.

In this study, the benchmark dataset was derived from dbPTM 3.0 [22] at http://dbptm.mbc.nctu.edu.tw/, a protein post-translational modifications database. For facilitating the description later, let us adopt Chou’s peptide formulation, which was used for investigating the HIV protease cleavage sites [23,24], the specificity of GalNAc-transferase [25], as well as signal peptide cleavage sites [26–29]. According to Chou’s scheme, a peptide with Pro (namely P in its single-letter code) or Lys (namely K) located at its center (Figure 2) can be expressed as:

(1) $$\{\begin{array}{c} P(P)=R_{-\xi }R_{-(\xi -1)}\cdots R_{-2}R_{-1}PR_{+1}R_{+2}\cdots R_{+(\xi -1)}R_{+\xi }\\ P(K)=R_{-\xi }R_{-(\xi -1)}\cdots R_{-2}R_{-1}KR_{+1}R_{+2}\cdots R_{+(\xi -1)}R_{+\xi } \end{array}$$

where the subscript, ξ, is an integer (*cf*. Figure 2), R_−ξ_ represents the ξ-th downstream amino acid residue from the center, R_ξ_ the ξ-th upstream amino acid residue, and so forth. Peptides **P**(ℙ) and **P**( 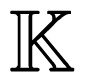) with the profile of Equation (1) can be further classified into the following categories:

(2) $$P(P)\in \{\begin{array}{l} \text{Pro}-\text{hydroxylated}\;\text{peptide},\;\;\;\text{if its center is}\;\text{a hydroxylation site}\\ \text{non}-\text{Pro}-\text{hydroxylated peptide},\;\;\;\text{otherwise} \end{array}$$

and:

(3) $$P(K)\in \{\begin{array}{l} \text{Lys}-\text{hydroxylated peptide},\;\;\;\text{if its center is a hydroxylation}\;\text{site}\\ \text{non}-\text{Lys}-\text{hydroxylated peptide},\;\;\;\text{otherwise} \end{array}$$

where ∈ represents “a member of” in the set theory.

As pointed out by a comprehensive review [30], there is no need to separate a benchmark dataset into a training dataset and a testing dataset for examining the performance of a prediction method if it is tested by the jackknife test or subsampling cross-validation test. Thus, the benchmark dataset for the current study can be formulated as:

(4) $$\{\begin{array}{l} S_{\text{HyP}}=S_{\text{HyP}}^{+}\cup S_{\text{HyP}}^{-}\\ S_{\text{HyL}}=S_{\text{HyL}}^{+}\cup S_{\text{HyL}}^{-} \end{array}$$

where 
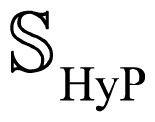
 is the benchmark dataset for studying hydroxyproline residues, 
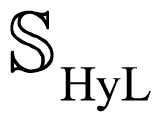
 the benchmark dataset for studying hydroxylysine residues, ⋃ the symbol for “union” in the set theory, $S_{\text{HyP}}^{+}$ contains the samples for the Pro-hydroxylated peptide only, $S_{\text{HyP}}^{-}$ contains the non-Pro-hydroxylated peptide only (*cf*. Equation (2)), $S_{\text{HyL}}^{+}$ contains the samples for the Lys-hydroxylated peptide only and $S_{\text{HyL}}^{-}$ contains the non-Lys-hydroxylated peptide only (*cf*. Equation (3)).

After some preliminary trials, we found that ξ = 6 was a good choice for the current study. Accordingly, each of the samples extracted from proteins in this study is actually a 2ξ +1 = 13 tuple peptide. If the upstream or downstream in a peptide sample was 3≤ ξ < 6, the lacking residues were filled with the dummy code, @. Furthermore, to reduce the redundancy and to avoid homology bias, those peptides were excluded from the benchmark datasets that had ≥ 80% pairwise sequence identity to any other in a same subset.

Finally, we obtained that the benchmark dataset, 
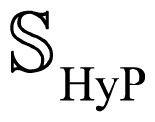
, contained 636 + 2699 = 3338 peptide samples, of which 636 were Pro-hydroxylated peptides belonging the positive subset $S_{\text{HyP}}^{+}$, and 2669 were non-Pro-hydroxylated peptides belonging to the negative subset, $S_{\text{HyP}}^{-}$; and that the benchmark dataset, 
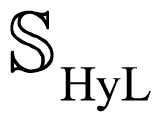
, contained 107 + 836 = 943 peptide samples, of which 107 were Lys-hydroxylated peptides belonging to the positive subset, $S_{\text{HyL}}^{+}$, and 836 were non-Lys-hydroxylated peptides belonging to the negative subset, $S_{\text{HyL}}^{-}$. For the reader’s convenience, the peptide sequences, as well as their hydroxylation or non-hydroxylation sites in proteins are given in the Supplementary Information, S1 and S2, for 
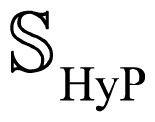
 and 
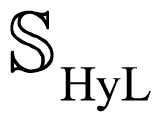
, respectively.

### 2.2. Feature Vector Construction

To develop a statistical method for predicting the attribute of peptides in proteins, one of the fundamental procedures was to formulate the peptide samples with an effective mathematical expression that could really reflect the intrinsic correlation with the desired target. To realize this, various feature vectors (see, e.g., [17,31–36]) were proposed to express peptides by extracting their different features into the pseudo amino acid composition [37,38] or Chou’s pseudo amino acid composition [39–41] or Chou’s PseAAC (pseudo amino acid composition) [42,43].

According to [21], the general form of PseAAC for a protein or peptide, **P**, can be formulated by:

(5) $$P={\left[\begin{array}{cccccc} \psi_{1} & \psi_{2} & \cdots & \psi_{u} & \cdots & \psi_{\Omega } \end{array}\right]}^{T}$$

where **T** is the transpose operator, while Ω is an integer to reflect the vector’s dimension. The value of Ω, as well as the components ψ_u_ (*u* = 1, 2, ···, Ω) in Equation (5) will depend on how to extract the desired information from the protein or peptide sequence. Below, let us describe how to extract the useful information from the benchmark datasets, 
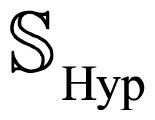
 and 
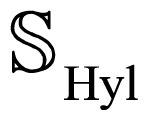
, to define the peptide samples via Equation (5).

Since each of the samples concerned is a 13-tuple peptide, Equation (1) can be simplified to a more convenient form given by:

(6) $$P={\text{R}}_{1}{\text{R}}_{2}\cdots {\text{R}}_{7}\cdots {\text{R}}_{12}{\text{R}}_{13}$$

where R_7_ = P or K, and R*_i_* (*i* = 1, 2, ··· , 13; *i* ≠ 7) can be any of the 20 native amino acids or the dummy code @, as defined above. Hereafter, let us use the numerical codes 1, 2, 3, ···, 20 to represent the 20 native amino acids according to the alphabetic order of their single letter codes and use 21 to represent the dummy amino acid, @. Accordingly, the number of possible different dipeptides will be 21 × 21 = 441, and the number of dipeptide subsite positions on the sequence of Equation (6) will be (13 − 2 + 1) = 12.

Now, let us introduce the following 441 × 12 matrix, ℤ, the so-called PSDP (position-specific dipeptide propensity) matrix [36], to define the component of Equation (5):

(7) $$\mathbb{Z}=\left[\begin{array}{cccc} Z_{1,1} & Z_{1,2} & \cdots & Z_{1,12}\\ Z_{2,1} & Z_{2,2} & \cdots & Z_{2,12}\\ \vdots & \vdots & \ddots & \vdots \\ Z_{441,1} & Z_{441,2} & \cdots & Z_{441,12} \end{array}\right]$$

where the element:

(8) $$Z_{i,j}=F^{+}({\text{D}}_{i}|j)-F^{-}({\text{D}}_{i}|j)\;\;\;(i=1,2,\cdots ,441;j=1,2,\cdots ,12)$$

and:

(9) $${\text{D}}_{1}=\text{AA},{\text{D}}_{2}=\text{AC},\cdots ,{\text{D}}_{21}=\text{A}@,\cdots ,{\text{D}}_{440}=@\text{Y},{\text{D}}_{441}=@@$$

In Equation (5)*F*^+^ (D*_i_* | *j*) is the occurrence frequency of the *i*-th dipeptide (*i* = 1,2, ···, 441) at the *j*-th subsite on the sequence of Equation (6) that can be easily derived from the positive dataset in the Supplementary Information S1 or S2; while *F*^−^ (D*_i_* | *j*) is the corresponding occurrence frequency, but derived from the negative dataset.

Thus, the peptide, **P**, of Equation (6) can be uniquely defined via the general form of PseAAC (*cf*. Equation (5)) with its dimension Ω = 12 and its *u*-th component given by:

(10) $$\psi_{u}=\{\begin{array}{cc} z_{1,u} & \text{when }{\text{R}}_{u}{\text{R}}_{u+1}=\text{AA}\\ z_{2,u} & \text{when }{\text{R}}_{u}{\text{R}}_{u+1}=\text{AC}\\ \vdots & \vdots \\ z_{21,u} & \text{when }{\text{R}}_{u}{\text{R}}_{u+1}=\text{A}@\\ \vdots & \vdots \\ z_{441,u} & \text{when }{\text{R}}_{u}{\text{R}}_{u+1}=@@ \end{array}\;\;\;(1\leq u\leq 12)$$

### 2.3. Prediction Algorithm

Suppose ℙ^+^ are the standard vectors or norms for the peptide sequences in the positive benchmark dataset, $S_{\text{HyP}}^{+}$ or $S_{\text{HyL}}^{+}$, and ℙ^−^ are those in the negative benchmark dataset, $S_{\text{HyP}}^{-}$ or $S_{\text{HyL}}^{-}$. Additionally, they are defined by:

(11) $$\{\begin{array}{c} P^{+}={\left[\begin{array}{cccccc} {\bar{\psi }}_{1}^{+} & {\bar{\psi }}_{2}^{+} & \cdots & {\bar{\psi }}_{u}^{+} & \cdots & {\bar{\psi }}_{\Omega }^{+} \end{array}\right]}^{T}\\ P^{-}={\left[\begin{array}{cccccc} {\bar{\psi }}_{1}^{-} & {\bar{\psi }}_{2}^{-} & \cdots & {\bar{\psi }}_{u}^{-} & \cdots & {\bar{\psi }}_{\Omega }^{-} \end{array}\right]}^{T} \end{array}$$

where:

(12) $$\{\begin{array}{l} {\bar{\psi }}_{u}^{+}=\frac{1}{N^{+}}\sum_{k=1}^{N^{+}}\psi_{u,k}^{+}\\ {\bar{\psi }}_{u}^{-}=\frac{1}{N^{-}}\sum_{k=1}^{N^{-}}\psi_{u,k}^{-} \end{array}\;\;\;(u=1,2,\cdots ,\Omega )$$

where *N*^+^ is the total number of Pro-hydroxylated peptides or Lys- hydroxylated peptides in the benchmark dataset, $S_{\text{HyP}}^{+}$ or $S_{\text{HyL}}^{+}$, and $\psi_{u,k}^{+}$ the *u*-th component for the *k*-th Pro-hydroxylated peptide or Lys-hydroxylated peptide in the PseAAC space (see Equations (5) and (10)); whereas *N*^−^ and $\psi_{u,k}^{-}$ have the same meanings, but are for the *K*-th non-Pro-hydroxylated peptide or non-Lys-hydroxylated peptide.

For a query peptide, **P**, as formulated by Equation (5), suppose 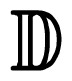
(**P**, ℙ^+^) is its similarity to the norm of hydroxylated peptides and 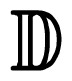
(**P**, ℙ^−^) its similarity to the norm of non-hydroxylated peptides, as formulated by:

(13) $$\{\begin{array}{l} D\left(P,P^{+}\right)=\sqrt{\sum_{u=1}^{\Omega }{\left(\psi_{u}-{\bar{\psi }}_{u}^{+}\right)}^{2}}\\ D\left(P,P^{-}\right)=\sqrt{\sum_{u=1}^{\Omega }{\left(\psi_{u}-{\bar{\psi }}_{u}^{-}\right)}^{2}} \end{array}$$

Thus, according to the discriminant function algorithm [24,44], the attribute of the query peptide, **P**, can be formulated as:

(14) $$P\in \{\begin{array}{ll} \text{Hydroxylated peptide}, & \text{if }D\left(P,P^{+}\right)<D\left(P,P^{-}\right)\\ \text{non}-\text{hydroxylated peptide}, & \text{otherwise} \end{array}$$

If there was a tie between 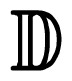
(**P**, ℙ^+^) and 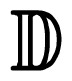
(**P**, ℙ^−^), the query peptide would be randomly assigned between the hydroxylated peptide and non-hydroxylated peptide categories. However, this kind of tie case rarely happened and actually never happened in our study.

The predictor established via the above procedures is called iHyd-PseAAC, where “i” stands for the first character of “identify”, “Hyd” for “hydroxylation” and “PseAAC” for the general form of the pseudo amino acid composition [21] used to formulate the peptide sequences.

**Figure 3.**
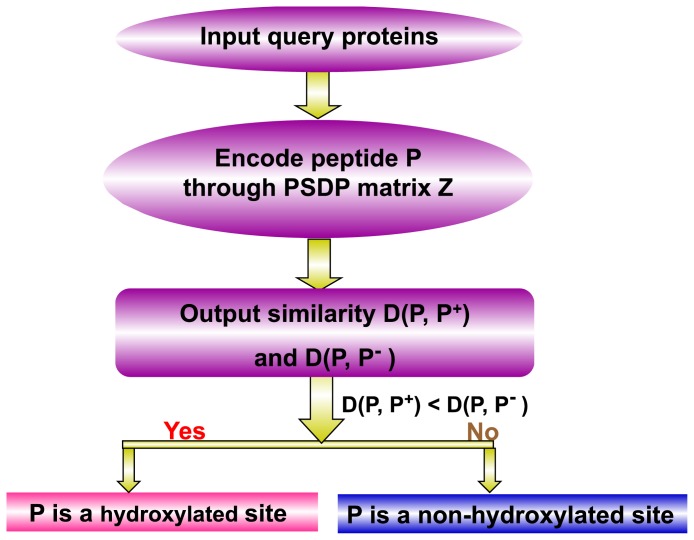
Flowchart to show the process of how the iHyd-PseAAC (identify hydroxylation pseudo amino acid composition) predictor works in identifying the hydroxylated sites in proteins. PSDP, position-specific dipeptide propensity.

A flowchart of the predictor is given in Figure 3 to illustrate how iHyd-PseAAC was working during the process of prediction.

## 3. Experimental Section

### 3.1. A Set of Metrics for Measuring Prediction Quality

To provide a more intuitive and easier-to-understand method to measure the prediction quality, the following set of four metrics based on the formulation used by Chou [26–28] in predicting signal peptides was adopted. According to Chou’s formulation, the sensitivity, specificity, overall accuracy, and Matthews correlation coefficient can be respectively expressed as [18,33,36,45]:

(15) $$\{\begin{array}{l} \text{Sn}=1-\frac{N_{-}^{+}}{N^{+}}\\ \text{Sp}=1-\frac{N_{+}^{-}}{N^{-}}\\ \text{Acc}=1-\frac{N_{-}^{+}+N_{+}^{-}}{N^{+}+N^{-}}\\ \text{Mcc}=\frac{1-\left(\frac{N_{-}^{+}+N_{+}^{-}}{N^{+}+N^{-}}\right)}{\sqrt{\left(1+\frac{N_{+}^{-}-N_{-}^{+}}{N^{+}}\right)\left(1+\frac{N_{-}^{+}-N_{+}^{-}}{N^{-}}\right)}} \end{array}$$

where *N*^+^ is the total number of the hydroxylated Pro-peptides (or Lys-peptides) investigated, while $N_{-}^{+}$ is the number of hydroxylated Pro-peptides (or Lys-peptides) incorrectly predicted as the non-hydroxylated Pro-peptides (or Lys-peptides); *N*^−^ is the total number of the non-hydroxylated Pro-peptides (or Lys-peptides) investigated, while $N_{+}^{-}$ is the number of the non-hydroxylated Pro-peptides (or Lys-peptides) incorrectly predicted as the hydroxylated Pro-peptides (or Lys-peptides).

According to Equation (15), we can easily see the following. When $N_{-}^{+}=0$, meaning none of the hydroxylated Pro-peptides (or Lys-peptides) was mispredicted to be a non-hydroxylated Pro-peptide (or Lys-peptides), we have the sensitivity Sn = 1; while $N_{-}^{+}=N^{+}$, meaning that all the hydroxylated Pro-peptides (or Lys-peptides) were mispredicted to be the non-hydroxylated Pro-peptides (or Lys-peptides), we have the sensitivity Sn = 0. Likewise, when $N_{+}^{-}=0$, meaning none of the non-hydroxylated Pro-peptides (or Lys-peptides) was mispredicted, we have the specificity Sn = 1; while $N_{+}^{-}=N^{-}$, meaning all the non-hydroxylated Pro-peptides (or Lys-peptides) were incorrectly predicted as hydroxylated Pro-peptides (or Lys-peptides), we have the specificity Sn = 0. When $N_{-}^{+}=N_{+}^{-}=0$, meaning that none of the hydroxylated Pro-peptides (or Lys-peptides) in the dataset $S_{\text{HyP}}^{+}$ (or $S_{\text{HyL}}^{+}$) and none of the hydroxylated Pro-peptides (or Lys-peptides) in $S_{\text{HyP}}^{-}$ (or $S_{\text{HyL}}^{-}$) was incorrectly predicted, we have the overall accuracy Acc = 1; while $N_{-}^{+}=N^{+}$ and $N_{+}^{-}=N^{-}$, meaning that all the hydroxylated Pro-peptides (or Lys-peptides) in the dataset $S_{\text{HyP}}^{+}$ (or $S_{\text{HyL}}^{+}$) and all the non-hydroxylated Pro-peptides (or Lys-peptides) in $S_{\text{HyP}}^{-}$ (or $S_{\text{HyL}}^{-}$) were mispredicted, we have the overall accuracy Acc = 0. The Matthews correlation coefficient (MCC) is usually used for measuring the quality of binary (two-class) classifications. When $N_{-}^{+}=N_{+}^{-}=0$, meaning that none of the hydroxylated Pro-peptides (or Lys-peptides) in the dataset $S_{\text{HyP}}^{+}$ (or $S_{\text{HyL}}^{+}$) and none of the non-hydroxylated Pro-peptides (or Lys-peptides) in $S_{\text{HyP}}^{-}$ (or $S_{\text{HyL}}^{-}$) was mispredicted, we have MCC = 1; when $N_{-}^{+}=N^{+}/2$ and $N_{+}^{-}=N^{-}/2$, we have MCC = 0, meaning no better than random prediction; when $N_{-}^{+}=N^{+}$ and $N_{+}^{-}=N^{-}$, we have MCC = 0, meaning total disagreement between prediction and observation. As we can see from the above discussion, it is much more intuitive and easier-to-understand when using Equation (15) to examine a predictor for its four metrics, particularly for its Mathew’s correlation coefficient. It is instructive to point out that the metrics as defined in Equation (15) are valid for single-label systems; for multi-label systems [34,46–48], a set of more complicated metrics should be used, as given in [49].

### 3.2. Jackknife Cross-Validation

How to properly test a predictor for its anticipated success rates is very important in objectively evaluating its quality and potential application value. Generally speaking, the following three cross-validation methods are often used to examine the quality of a predictor and its effectiveness in practical application: the independent dataset test, the subsampling or the *K*-fold (such as 5-, 7- or 10-fold) crossover test and the jackknife test [50]. However, as elaborated by a penetrating analysis in [51], considerable arbitrariness exists in the independent dataset test. Furthermore, as demonstrated in [52], the subsampling (or *K*-fold crossover validation) test cannot avoid arbitrariness either. The jackknife test is the least arbitrary, which can always yield a unique result for a given benchmark dataset. Therefore, the jackknife test has been widely recognized and increasingly utilized by investigators to examine the quality of various predictors (see, e.g., [32,53–62]). Accordingly, in this study, the jackknife test was also adopted to evaluate the accuracy of the current predictor. Listed in Table 1 are the jackknife test results obtained by iHyd-PseAAC on the benchmark datasets of Supplementary Information S1 and the benchmark dataset of Supplementary Information S2, respectively.

To further demonstrate our predictor, the jackknife test was conducted on two more stringent benchmark datasets given in Supplementary Information S3 and S4, where none of the included sequences has more than 40% pairwise sequence identity with any other. The results thus obtained are listed in Table 2.

It is interesting to see by comparing the two tables that the rates of Acc and MCC are about the same in both cases. Although the rates of Sn in Table 2 are somewhat lower than those in Table 1, the rates of Sp in Table 2 are higher than those in Table 1. Accordingly, the success rates as measured by the four metrics in Equation (15) are basically about the same without dropping down significantly from using an 80% cutoff benchmark dataset to a 40% cutoff one, clearly indicating that iHyd-PseAAC is a useful predictor validated by rigorous cross-validation.

### 3.3. Test by Public Database

Moreover, from the Swiss-Prot database, we retrieved all those proteins whose hydroxylated sites were experimentally validated. After excluding those with a length less than 50 amino acids, we obtained 156 hydroxyproline proteins and 31 hydroxylysine proteins, respectively. Their codes and hydroxylated sites are given in Supplementary Information S5 and S6, respectively. The predicted results by iHyd-PseAAC on these real proteins are given in Table 3, from which we can see that the overall success rates thus obtained are quite consistent with those derived by the cross-validation on the benchmark datasets, as shown in Tables 1 and 2, fully indicating that iHyd-PseAAC is not only a valid predictor, but also may become a very useful high throughput toll for practical applications in this area.

## 4. Conclusions

As we can see from Table 1, the overall accuracies for the hydroxyproline and hydroxylysine cases are 80.57% and 83.56%, which are higher than 76.0% and 82.1%, the corresponding rates reported by Hu *et al*. [11]. At first glance, the value of MCC seems relatively low. Actually, as mentioned in Section 3.1, different from Acc, whose score is between 100% and 0%, the score for MCC is between one and −1, with zero meaning no better than random prediction. Accordingly, the MCC rate of 0.50–0.51 is generally deemed as a quite decent result. Particularly, the benchmark dataset in the current system is very imbalanced, which contains 636 hydroxylated peptides and 2669 non-hydroxylated peptides for proline, which also may lower the MCC rate. The same is true for the case of hydroxylation.

Particularly, no web-server was provided for the method in [11], and hence, its application value is quite limited. Actually, so far, no web-server whatsoever has been provided in this area. As pointed out in [63] and emphasized in a series of recent publications (see, e.g., [16–18,20,42,45]), one of the keys in developing a practically more useful prediction method is to establish a user-friendly and publicly accessible web-server. In view of this, the web-server for iHyd-PseAAC has been established, which can be freely accessed at http://app.aporc.org/iHyd-PseAAC/.

Furthermore, for the convenience of the vast majority of biologists and pharmaceutical scientists, below, let us provide a step-by-step guide to show how the users can easily get the desired result by using iHyd-PseAAC without the need to follow the complicated mathematical equations presented in this paper just for its integrity.

## 5. The User Guide for the Web-Server iHyd-PseAAC

**Figure 4.**
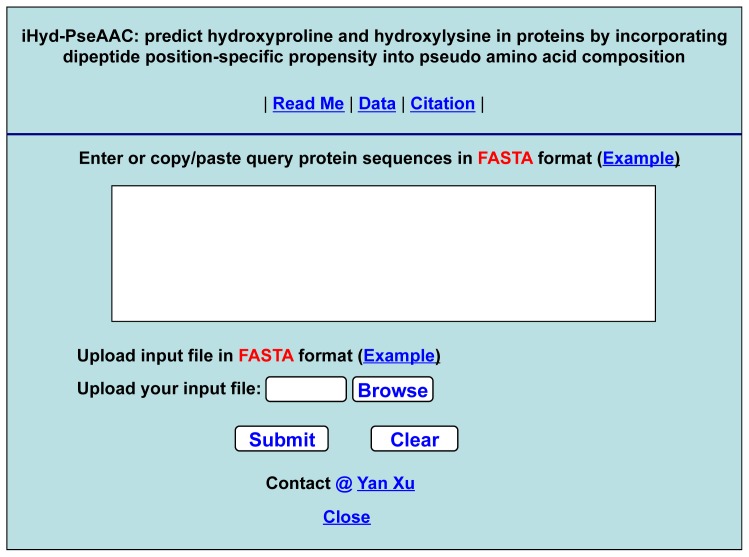
The top-page of the web-server, iHud-PseAAC, at http://app.aporc.org/iHyd-PseAAC/.

Step 1. Open the web-server at the site at http://app.aporc.org/iHyd-PseAAC/, and you will see the top page of the predictor on your computer screen, as shown in Figure 4. Click on the “Read Me” button to see a brief introduction about the iHyd-PseAAC predictor and the caveat when using it.
Step 2. Either type or copy/paste the query protein sequences into the input box at the center of Figure 4. The protein sequences should be in FASTA format. The input examples can be seen by clicking on the “Example” button right above the input box.
Step 4. Click on the “Citation” button to find the relevant paper that documents the detailed development and algorithm of iHyd-PseAAC.
Step 5. Click on the “Data” button to download the benchmark dataset used to train and test the iHyd-PseAAC predictor.

## Supplementary Information

Supplementary Information S1. The positive dataset, $S_{\text{HyP}}^{+}$, and negative dataset, $S_{\text{HyP}}^{-}$, contain 636 hydroxyproline peptide fragments and 2699 non-hydroxyproline peptide fragments, respectively. None of the sequences included has ≥ 80% pairwise sequence identity with any other in the same subset.

Supplementary Information S2. The positive dataset, $S_{\text{HyL}}^{+}$, and negative dataset, $S_{\text{HyL}}^{-}$, contain 107 hydroxylysine peptide fragments and 836 non-hydroxylysine peptide fragments, respectively. None of the sequences included has ≥ 80% pairwise sequence identity with any other in the same subset.

Supplementary Information S3. The positive dataset, $S_{\text{HyP}}^{+}$, and negative dataset, $S_{\text{HyP}}^{-}$, contain 306 hydroxyproline peptide fragments and 1035 non-hydroxyproline peptide fragments, respectively. None of the sequences included has ≥ 40% pairwise sequence identity with any other in the same subset.

Supplementary Information S4. The positive dataset, $S_{\text{HyL}}^{+}$, contains 44 hydroxylysine peptide fragments, and the negative dataset, $S_{\text{HyL}}^{-}$, contains 528 non-hydroxylysine peptide fragments. None of the sequences included has ≥ 40% pairwise sequence identity with any other in the same subset.

Supplementary Information S5. The 156 experimentally validated hydroxyproline proteins and their hydroxylated sites were retrieved from the Swiss-Prot database.

Supplementary Information S6. The 31 experimentally validated hydroxylysine proteins and their hydroxylated sites were retrieved from the Swiss-Prot database.

## Figures and Tables

**Table 1.** The jackknife test results by the new predictor on the benchmark datasets in the Supplementary Information S1 and S2. HyP, hydroxyproline; HyL, hydroxylysine; Sn, sensitivity; Sp, specificity; Acc, accuracy; MCC, Matthews correlation coefficient.

Benchmark dataset a	Sn (%)	Sp (%)	Acc (%)	MCC
Supplementary Information S1 for HyP	80.66	80.54	80.57	0.51
Supplementary Information S2 for HyL	87.85	83.01	83.56	0.50

a None of the sequences included has more than 80% pairwise sequence identity with any other.

**Table 2.** The jackknife test results by the iHyd-PseAAC predictor on the benchmark datasets in Supplementary Information S3 and S4.

Benchmark dataset a	Sn (%)	Sp (%)	Acc (%)	MCC
Supplementary Information S1 for HyP	70.68	89.03	78.42	0.52
Supplementary Information S2 for HyL	79.04	86.37	83.12	0.51

a None of sequences included has more than 40% pairwise sequence identity with any other.

**Table 3.** The overall success rates in identifying hydroxylated sites for the proteins retrieved from the Swiss-Prot database.

Hydroxylated type	Sn (%)	Sp (%)	Acc (%)
Proline	71.2	79.3	75.3
Lysine	72.7	80.6	76.8
